# The genome sequence of the avian vampire fly (*Philornis downsi*), an invasive nest parasite of Darwin’s finches in Galápagos

**DOI:** 10.1093/g3journal/jkab414

**Published:** 2021-12-08

**Authors:** Melia G Romine, Sarah A Knutie, Carly M Crow, Grace J Vaziri, Jaime A Chaves, Jennifer A H Koop, Sangeet Lamichhaney

**Affiliations:** 1 School of Biomedical Sciences, Kent State University, Kent, OH 44240, USA; 2 Department of Ecology and Evolutionary Biology, University of Connecticut, Storrs, CT 06269, USA; 3 Institute for Systems Genomics, University of Connecticut, Storrs, CT 06269, USA; 4 Department of Biological Sciences, Northern Illinois University, DeKalb, IL 60115, USA; 5 Department of Biology, San Francisco State University, San Francisco, CA 94132, USA; 6 Colegio de Ciencias Biológicas y Ambientales, Universidad San Francisco de Quito, Quito 170901, Ecuador; 7 Department of Biological Sciences, Kent State University, Kent, OH 44240, USA

**Keywords:** avian vampire fly genome, parasitic invasion, Galápagos, insecticide resistance, Darwin’s finches

## Abstract

The invasive avian vampire fly (*Philornis downsi*, Diptera: Muscidae) is considered one of the greatest threats to the endemic avifauna of the Galápagos Islands. The fly larvae parasitize nearly every passerine species, including Darwin’s finches. Most *P. downsi* research to date has focused on the effects of the fly on avian host fitness and mitigation methods. A lag in research related to the genetics of this invasion demonstrates, in part, the need to develop full-scale genomic resources with which to address further questions within this system. In this study, an adult female *P. downsi* was sequenced to generate a high-quality genome assembly. We examined various features of the genome (*e.g.*, coding regions and noncoding transposable elements) and carried out comparative genomics analysis against other dipteran genomes. We identified lists of gene families that are significantly expanding or contracting in *P. downsi* that are related to insecticide resistance, detoxification, and counter defense against host immune responses. The *P. downsi* genome assembly provides an important resource for studying the molecular basis of successful invasion in the Galápagos and the dynamics of its population across multiple islands. The findings of significantly changing gene families associated with insecticide resistance and immune responses highlight the need for further investigations into the role of different gene families in aiding the fly’s successful invasion. Furthermore, this genomic resource provides a necessary tool to better inform future research studies and mitigation strategies aimed at minimizing the fly’s impact on Galápagos birds.

## Introduction

**Figure 1:**
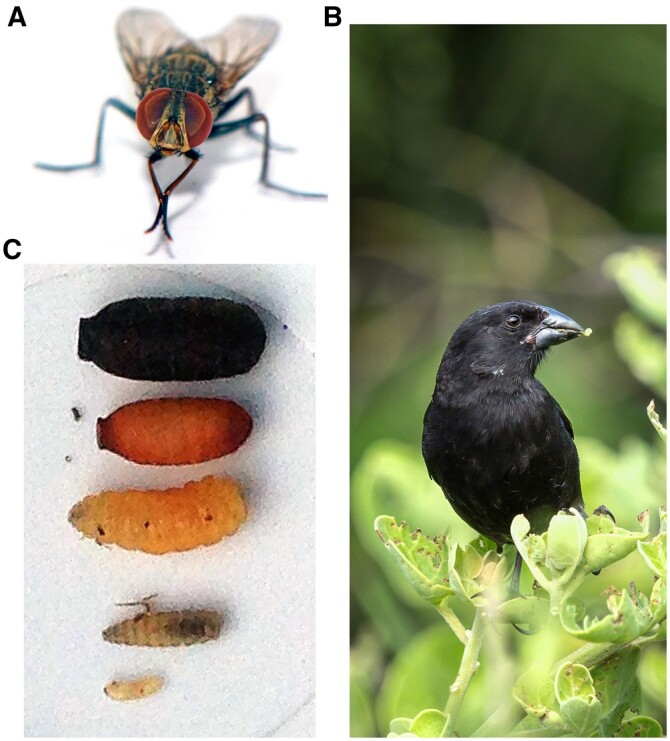
The avian vampire fly, *P. downsi* (A), parasitizes many endemic bird species of the Galápagos Islands, including the medium ground finch, *Geospiza fortis* (B). The fly is parasitic in its larval forms (C, bottom three) residing primarily in the nesting material. The larvae feed by chewing through the skin and feeding on the secreted blood and other fluids of avian hosts. Larvae then pupate (C, second from top) in the nest material and enclose (C, top) as adult flies. Photos courtesy of Sam Rowley (A), Kelly Ballantyne (B), and Sabrina McNew (C).

The invasive avian vampire fly (*Philornis downsi*, Diptera: Muscidae) (Figure  1A) is considered among the greatest threats to the unique and endemic avifauna of the Galápagos Islands (Causton *et al.* 2013). In its larval form, the fly parasitizes eleven species of Darwin’s finches as well as nearly every other Galápagos passerine species (Causton *et al.* 2013; Fessl *et al.* 2018; Figure  1). The larvae are obligate nest ectoparasites that feed on the blood and other fluids of nestling and brooding adult birds (Fessl and Tebbich 2002). The impact of parasitism by the fly has been severe in some populations of birds in Galápagos and while the effects are variable, some studies have reported near-total nest failure rates due to parasitism (Dudaniec *et al.* 2007; Koop *et al.* 2011, 2013; O’Connor *et al.* 2014; Knutie *et al.* 2016; Heimpel *et al.* 2017; Addesso *et al.* 2020). The fly has also been implicated in the decline of the medium tree finch (*Camarhyncus pauper*), the warbler finch (*Certhidia olivacea*), and the mangrove finch (*Camarhyncus heliobates*) (Dvorak *et al.* 2004; Grant *et al.* 2005; Cunninghame *et al.* 2017; Peters and Kleindorfer 2018; Bulgarella *et al.* 2019). Furthermore, the potential for population-level declines of even relatively prominent bird species, *e.g.*, the medium ground finch, have also been demonstrated using predictive models (Koop *et al.* 2016). 

The genus *Philornis* includes approximately 50 species found primarily in the Neotropics and North America (Dodge 1955, 1963; Spalding *et al.* 2002; Dudaniec and Kleindorfer 2006; Couri *et al.* 2007). Several *Philornis* species are found in mainland Ecuador (Bulgarella *et al.* 2015, 2017), but *P. downsi* remains the only recorded species of the genus present, to date, in the Galápagos. *Philornis* *downsi* is thought to have been introduced from mainland Ecuador, though the full pathway of invasion is not yet known (Fessl *et al.* 2018). Preliminary population genetics studies show that *P. downsi* in the archipelago have a high degree of relatedness relative to those on the mainland, which supports the hypothesis of a relatively recent invasion, and also the possibility of continued gene flow between populations in the Galápagos (Dudaniec *et al.* 2008; Koop *et al.* 2021).

Despite the robust number of studies that explored the effects of these flies on hosts in the Galápagos (Causton *et al.* 2013, 2019), questions remain about the underlying ecological and evolutionary mechanisms of their successful invasion in the Galápagos. This knowledge gap demonstrates the need to develop a full-scale genomic resource of *P. downsi* as it provides a critical knowledge base from which to explore these questions, similar to other parasites of concern (*e.g.*, Scott *et al.* 2020). In this study, we first generated a high-quality draft genome of *P. downsi*. Furthermore, we carried out comparative genomic analyses with other published dipteran genomes that identified evidence of significantly expanded or contracted gene families associated with insecticide resistance, detoxification, and possible counter defense against host immune responses. The identification of these gene families serves as the first step toward investigations of the fly’s ability to rapidly evolve traits associated with its successful invasion in the Galápagos. This genomic resource provides a necessary tool to better inform future research studies and mitigation strategies aimed at minimizing the fly’s impact on the birds of the Galápagos.

## Materials and methods

### Sampling and DNA extraction for genome sequencing

*Philornis downsi* were collected in Jardín de las Opuntias on San Cristobal Island, Galápagos, Ecuador (−0.9491651°, −89.5528454°) in March-April of 2019. Adult flies were reared from pupae collected from the nests of small ground finches (*Geospiza fuliginosa*). When nests were empty, either because nestlings died or fledged, all larvae and pupae were extracted from the nest and placed in a falcon tube with a modified lid that allowed airflow. After flies eclosed from their pupal case, they were placed in a freezer (−20°C) to immobilize them, then preserved in 95% ethanol. Preserved flies were transported to the University of Connecticut, then shipped to Northern Illinois University for further processing. DNA was extracted using DNeasy Blood and Tissue Kit (Qiagen, Valencia, CA, USA) on whole fly samples after wings and legs were removed using forceps. All samples were treated with Monarch^®^ RNase A (New England Biolabs) to remove RNA from genomic DNA samples. Multiple samples were extracted and those with low yields of DNA (<1 µg of total yield) or fragmentation (samples that showed multiple bands on agarose gel) were discarded from further processing. Quantification of double-stranded DNA was done using QuBit^®^ and samples were run on an agarose gel to assess fragmentation. Finally, the sample from a single female individual with the highest DNA yield of molecular weight (>100 kb) was chosen for library preparation and genome sequencing.

### Estimate of genome size and ploidy

We prepared a 10X Chromium GEM sequencing library using the extracted DNA from a single female individual according to the manufacturer’s recommended genome assay linked-read workflow. The library was sequenced on the Illumina Novaseq platform using NovaSeq 6000 Reagent Kit to generate paired-end 150 bp reads from ∼350 bp average insert size fragments. We used a k-mer based approach to estimate genome size, heterozygosity, and repeat content from unprocessed short-sequencing reads (Vurture *et al.* 2017). We also used the k-mer distribution to extract heterozygous k-mer pairs to assess the ploidy level in *P. downsi* (Ranallo-Benavidez *et al.* 2020)*.* These estimates of genome size and ploidy were then used to choose parameters for the downstream genome assembly pipeline.

### Genome assembly and annotation

The 10X Chromium linked reads were used to generate a reference genome using Supernova.v.2.1.1 (Weisenfeld *et al.* 2017) with default parameters. The genome contiguity statistics such as scaffold N_50_ and the total number of scaffolds were calculated using custom scripts. We further compared the draft genome assembly against a set of conserved genes in Diptera using BUSCO version 4.0.6 (Waterhouse *et al.* 2017) to assess gene-space completeness. The annotation of the *P. downsi* genome was done using the Maker (version 3.01.03) annotation pipeline (Cantarel *et al.* 2008). The parameters and configuration files used to run Maker are provided on our lab GitHub page (see “Data availability”). We generated protein homology evidence using publicly available proteome datasets from two highly curated dipteran genomes, *Musca domestica* (Scott *et al.* 2014) and *Drosophila melanogaster* (Adams *et al.* 2000), downloaded from the Ensembl metazoa database (Cunningham *et al.* 2019). We used *D. melanogaster*, version BDGP6.32, and *M.* *domestica* version MdomA1 from the Ensembl metazoa database for these annotations. In addition, we used a built-in pipeline in MAKER (Augustus) to generate *ab* *initio* gene prediction models to annotate the *P. downsi* genome.

### Transposable elements in *Philornis downsi*

We combined homology-based and *de novo* approaches using Repeatmasker version 4.1.0 (Hubley and Green 2013; Flynn *et al.* 2019) to characterize transposable elements (TEs) in *P. downsi*. We used two different sets of repeat libraries, (1) a reference repeat library downloaded from the Repbase database (release 20190301) (Bao *et al.* 2015) and (2) a *de novo* custom-built species-specific repeat library generated for *P. downsi* using RepeatModeler version 2.0.1 (Flynn *et al.* 2019). The usage of the *de novo* species-specific repeat library increased the accuracy of detection and annotation of TEs. For an unbiased comparison of repeat landscapes among all species, we used similar approaches to detect TEs in the genomes of house fly (*M.* *domestica*, NCBI accession no. GCA_002191195.1), stable fly (*Stomoxys calcitrans*, NCBI accession no. GCA_001015335.1), tsetse fly (*Glossina morsitans*, NCBI accession no. GCA_001077435.1), Mediterranean fruit fly (*Ceratitis capitata*, NCBI accession no. GCA_000347755.4), fruit fly (*D. melanogaster*, NCBI accession no. GCA_000001215.4), and yellow fever mosquito (*Aedes aegypti*, NCBI accession no. GCA_002204515.1), together with an outgroup, postman butterfly (*Heliconius melpomene*, NCBI accession no. GCA_000313835.2).

### Orthologs to other dipteran genomes

We inferred orthogroups and orthologs by comparative analysis of genomes from *P. downsi* and six additional dipteran species (*M. domestica*, *S. calcitrans*, *G. morsitans*, *C. capitata*, *D. melanogaster*, and *A. aegypti*), together with an outgroup, *H. melpomene.* This analysis used the draft proteome of *P. downsi* generated from our genome annotation pipeline described above. Complete proteomes of each of the remaining seven species were downloaded from the Ensembl database (Cunningham *et al.* 2019). The orthologs inference was carried out using OrthoFinder version 2.5.2 with default parameters (Emms and Kelly 2015). Only the longest isoform of each gene was used as an inference for orthologs.

### Gene family evolution

To explore the evolution of gene families in *P. downsi* relative to other Dipterans, we examined the rooted species tree generated by Orthofinder. Orthofinder first infers a gene tree for each orthogroup using DendroBLAST (Kelly and Maini 2013) and then infer a species tree from the set of gene trees using STAG (Emms and Kelly 2018) and finally roots the species tree using STRIDE (Emms and Kelly 2017). STAG and STRIDE can leverage information from multigene families to build a robust species tree rather than relying only on sets of single-copy orthologs (Emms and Kelly 2015).

We used this phylogeny to analyze the significant changes in gene family size across lineages using a maximum likelihood approach CAFÉ, version 4.2.1 with default parameters (De Bie *et al.* 2006). CAFÉ uses a birth and death process to model gene gain and loss across a phylogenetic tree and assesses the distribution of family size under this model to estimate the significance of differences in gene family size across taxa (*P* < 0.01). The separate birth (λ) and death (μ) rates were estimated for this dataset using the lambda (mu) command.

## Results and discussion

### Genome sequencing and assembly

A DNA library was prepared from a single adult female *P. downsi* fly using the 10× Chromium linked-read approach and sequenced on the Illumina platform to generate paired-end 150-bp reads, resulting in ∼479 million read pairs (∼72 Gb of raw sequence data). We first generated a k-mer distribution based on these short-sequencing reads for preliminary characterization of genome structure in *P. downsi* (Supplementary Table S1 and Supplementary Figure S1) that further allowed us to make informed decisions on parameters needed for building a reference genome. We also utilized the k-mer distribution to estimate the ploidy level in *P. downsi*, which indicated it to be a diploid species (Supplementary Figure S2).

Based on k-mer based genome size estimate (782.6 Mb) we had ∼91× sequence coverage for generating the draft *de* *novo* genome assembly. The total estimated length of the draft genome assembly was 971.6 Mb. The assembly contained 41,176 total scaffolds (minimum 1000 bp to maximum 8.6 Mb) with scaffold N_50_ of 1.3 Mb. We used blastn (BLAST version 2.9.0+) with the draft genome both as “database” and “query” to assess sequence similarity among different scaffolds. All 100% identical scaffolds were flagged as “duplicated,” and these 3954 scaffolds (total 19 Mb) were removed from downstream analysis. These scaffolds were all short in length (<5 kb) and possibly the result of misassembly.

Gene-space completeness was assessed using BUSCO version 4.0.6. Among 3258 genes highly conserved across Diptera, 3147 (95.8%) complete BUSCO genes were identified (3059 single copies and 88 duplicated) in the *P. downsi* draft genome assembly. Partial sequences of 71 genes were identified (2.2%) and only 67 (2.0%) were missing, which indicated a high degree of gene-space completeness in the *P. downsi* genome.

### Genome annotation

We combined protein homology-based evidence and *ab* *initio* gene prediction models using the MAKER genome annotation pipeline (Cantarel *et al.* 2008) which identified 15,774 protein-coding genes in the *P. downsi* genome. The genome annotation of *P. downsi* was compared against two closely related Muscidae species (1) *M. domestica*, annotation version *MdomA1* and (2) *S.* *calcitrans*, annotation version *ScalU1* from Ensembl metazoa database. The number of genes annotated in *P. downsi* (15,774) was slightly higher compared to *M. domestica* (14,402) and *S. calcitrans* (14,078) (Table  1). The average length of genes in *P. downsi* was significantly smaller than two other Muscidae species, while the average length of exons was similar. These results indicate that the introns in *P. downsi* are relatively compact compared to other Muscidae species. However, this could also be an indication that there are missing sequences in the assembled genome of *P. downsi* causing these differences in comparison with other Muscidae genomes.

**Table 1:** Statistics of genomic features among three fly genomes

Genome statistics	*P. downsi*	*M. domestica* (*v. MdomA1*)	*S. calcitrans* (*v. ScalU1*)
Genome size (Gb)	0.97	0.76	0.97
Total genes	15,774	14,402	14,078
Avg. gene length (bp)	4,789	15,078	25,802
Total exons	56,595	109,373	145,506
Avg. exon length (bp)	367	441	452
Number of exons per gene	3.59	7.59	10.34

Total exons and the average number of exons per gene were also lower in *P. downsi*. However, the number of exons in *P. downsi* may have been underestimated because we do not currently have information on alternative splicing and the number of isoforms for each gene in *P. downsi* due to lack of tissue-specific transcriptome data in this study. We aimed to carry out RNA sequencing of *P. downsi* tissues to build transcriptomes that would generate better experimental evidence of annotated gene models. Travel restrictions to our field sites in Galápagos during the COVID-19 pandemic hindered our ability to obtain these “RNA-quality” tissues. We acknowledge this limitation in the current study and aim to revise genome annotations in a future version of the *P. downsi* genome using this approach. Shorter average gene length may also reflect higher genome fragmentation in *P. downsi* compared to the other two species that have relatively contiguous genomes.

### Transposable elements in *Philornis downsi*

Mobile TEs are key features of eukaryotic genomes, being major determinants of genome size variation (Kapusta *et al.* 2017; Lamichhaney *et al.* 2021), and important contributors to the evolution of an organism (Pourrajab and Hekmatimoghaddam 2021). We characterized the TEs in the *P. downsi* genome using homology-based (Hubley and Green 2013) and *de-novo* approaches (Flynn *et al.* 2019). More than half of the genome (51.7%) of *P. downsi* consists of TEs (Supplementary Table S2). Among these sequences, 9.3% of the genome are retroelements (7.7% LINEs and 1.6% LTRs) and 23.4% are DNA transposons. Short interspersed nuclear elements (SINEs), a major category of retroelements, were not detected in the *P. downsi* genome.

**Figure 2:**
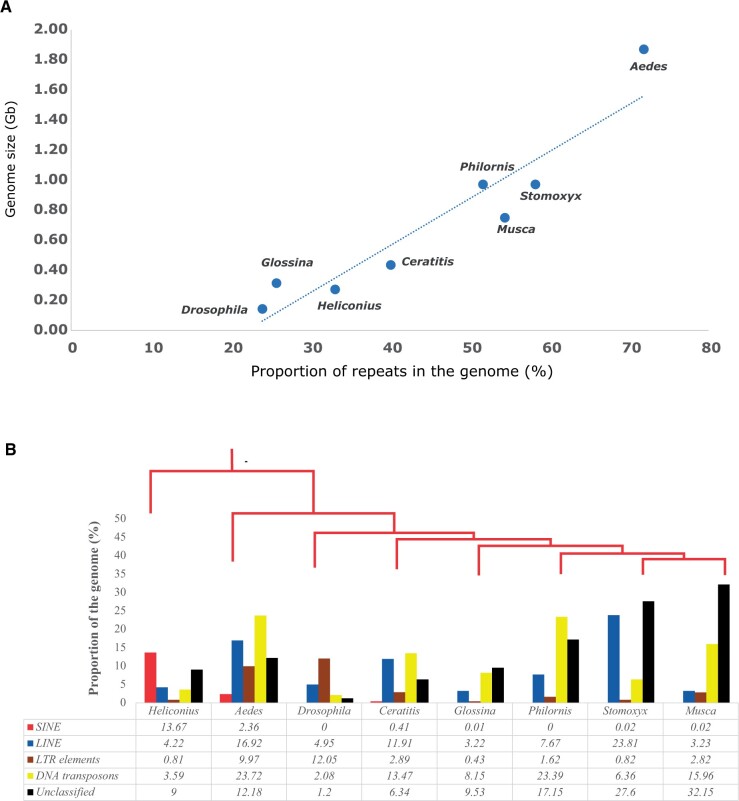
Landscape of transposable elements in *P. downsi*. (A) Comparison of repeat content and genome size across Diptera and its outgroup. (B) Repeat statistics on various classes of transposable elements across dipteran genomes.

Transposable elements are known to be highly correlated with genome size across the tree of life (Kidwell 2002; Lynch 2007), and our results across various dipteran genomes are consistent with this pattern (Figure  2A). *Philornis* *downsi* had the highest proportion of DNA transposons (23.4%) among all taxa analyzed. In comparison, only 6.36% of the *S. calcitrans* genome and 15.96% of the *M. domestica* genome consisted of DNA transposons. Although *P. downsi* and *S. calcitrans* have similar genome sizes, *S. calcitrans* had slightly higher amounts of repeat content (58.3%), compared to *P. downsi* (51.7%) (Figure  2B). *Stomoxys* *calcitrans* had higher amounts of long interspersed nuclear elements (LINEs) (23.8%), compared to *P. downsi* (7.7%).

Transposable elements are typically noncoding sequences that can insert themselves in various places of the genome, often with neutral or deleterious phenotypic consequences (Bourque *et al.* 2018). The role of TEs, as well as their evolution across insect genomes, is still an area of major research, but they are thought to be important drivers of genomic architecture depending on the location of the genome to which they insert themselves (*i.e.*, coding *vs* noncoding regions). Furthermore, TEs may also be a critical mechanism of adaptive evolution, as has been shown in an invasive ant species (Schrader *et al.* 2014). Analysis of TEs in *P. downsi* and across other dipteran genomes showed a strong positive correlation between genome size and repeat content (Figure  2A), consistent with similar findings across other taxa (Lynch 2007; Lamichhaney *et al.* 2021). Interestingly, *P. downsi* had a higher number of DNA transposons (Class II TEs) than any other compared genome, including *M. domestica*, *S. calcitrans*, and *G. morsitans*. While long terminal repeat (LTR) transposons, LINEs, and SINEs were present in the species studied, *P. downsi* had no SINEs, a finding consistent with a study by Petersen and colleagues (Petersen *et al.* 2019) showing that SINEs contribute less than 1% to the TE content of dipterans. However, it is important to note that some SINEs may be present in *P. downsi* but are currently masked as unclassified. Future research should explore the role of TEs, especially DNA transposons, in aiding the invasion of *P. downsi* to the Galápagos.

### Orthologs to other dipteran genomes and outgroup *Heliconius*

**Figure 3:**
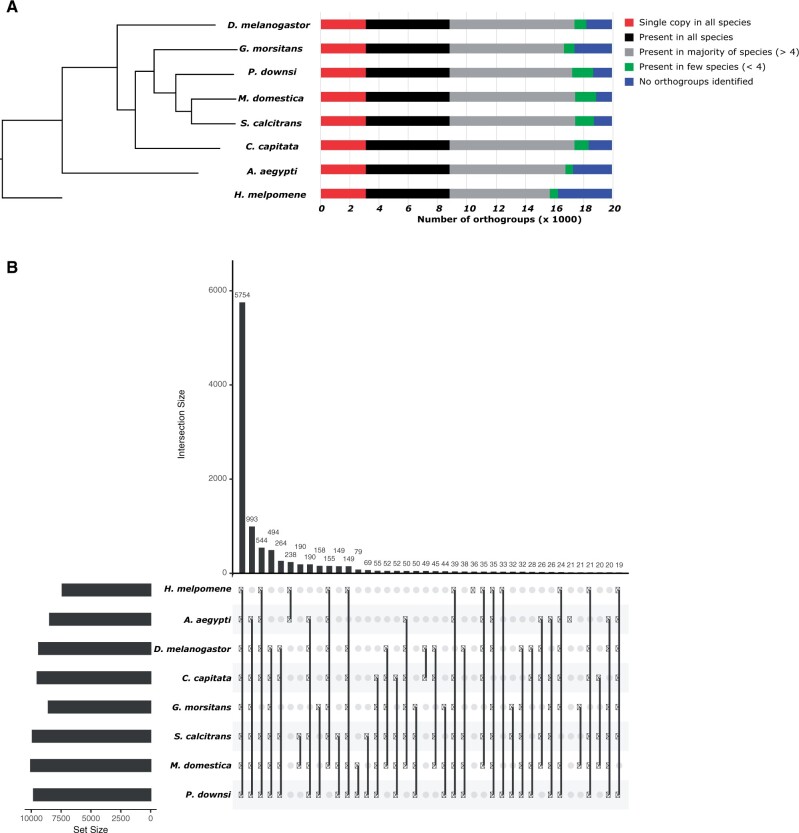
Orthogroups in *P. downsi*. (A) Phylogenetic relationship between *P. downsi* and other seven dipteran species, estimated using alignments from 3069 orthogroups had singly copy orthogroups in each species. Horizontal bars for each species show number of orthogroups that are single-copy orthologs in all species, present in all species, present in most species, present in few species, and unique to the species. (B) Number of shared orthologs among all species. Each vertical line connecting dots indicates the number of gene families (in the *y*-axis) that are shared among species analyzed. Certain shared gene families among species or unique gene families within a species had low count (<19), hence not included in the figure.

Comparative genomics analysis of 113,047 protein sequences from *P. downsi* and seven other species (listed above) identified 11,112 orthogroups. A total of 95,567 proteins (out of 113,047, 84.5%) were assigned to these orthogroups. The mean size of an orthogroup was 8.8 genes/species, and 3069 orthogroups had single-copy genes in each species. A total of 5754 orthogroups were shared among all eight species. Orthogroups present in fewer than four species ranged from 496 in *A. aegypti* to 1445 in *M. domestica* (Figure  3A). The number of unique orthologs in each species was mostly consistent with their phylogenetic relationships (*e.g.*, *A. aegypti* and the outgroup *H. melpomene* had the highest number of unique orthogroups). However, *G. morsitans* had the highest number of unique orthologs among the rest of the species compared. The number of shared orthologs among each pair of species is presented in Supplementary Table S3. The number of orthologs identified in *P. downsi* is consistent with other published dipteran genomes (Figure  3A and Supplementary Table S3), indicating no major bias in the genome assembly and annotation pipeline used in this study.

Within *P. downsi*, 13,706 out of 15,774 (86.9%) annotated genes were assigned to orthogroups. We expect the remaining missing genes to either be the most recently evolved orphan genes in the branch leading to the *P. downsi* lineage or the consequence of a lack of inclusion of enough closely related species of *P. downsi* in the analysis. The distribution of these unique genes in the *P. downsi* genome is random and does not show specific clustering patterns across various locations of the genome.

We also compared the number of pairwise orthogroups that are uniquely shared among all eight species (Figure  3B). A total of 993 orthogroups were unique to Diptera (after excluding the outgroup *H. melpomene*), 79 orthogroups were shared only between *M. domestica* and *P. downsi* and 12 putative gene families were unique to only *P. downsi.* These 12 gene families consisted of 27 genes and the great majority had the best BLAST hits against “uncharacterized” or “hypothetical” proteins in other related species (Supplementary Table S4). This result indicates that these gene families that appeared “unique” in *P. downsi* are likely false predictions as we do not have experimental evidence (*e.g.*, transcriptome data) for the gene models in *P. downsi* for accurate identification of its orthologs in other species compared.

### Gene family evolution

Across 11,112 orthogroups identified, 101 gene families showed evidence of significant expansion or contraction (*P* < 0.01) across the phylogenetic tree. Twenty-five of these 101 gene families were identified in the branch leading to *P. downsi*. We filtered these 25 putative gene families by excluding: (1) families where all genes were annotated as “uncharacterized” or “hypothetical” protein in all species, and (2) genes that were annotated from genome scaffolds in *P. downsi* that had high sequence similarity (>95%, putative duplicated scaffolds). Following filtration, 13 gene families showed evidence of significant expansion or contraction (*P* < 0.01) in *P. downsi* (Table  2).

**Table 2:** List of significantly expanding/contracting (*P* < 0.01) gene families in *P. downsi*

S.No	Ortholog ID	Gene family name	*P*-value
1	OG0000008	Fibrinogen C-terminal domain-containing protein^a^	<0.0001
2	OG0000009	Histone 2B^b^	<0.0001
3	OG0000014	Histone H1^b^	<0.0001
4	OG0000015	Cuticular protein^b^	<0.0001
5	OG0000017	Scp domain-containing protein^a^	<0.0001
6	OG0000040	Cytochrome P450^b^	<0.0001
7	OG0000044	Sant-like DNA-binding domain^b^	<0.0001
8	OG0000055	Protein takeout^b^	<0.0001
9	OG0000057	Nuclease HARBI1^a^	<0.0001
10	OG0000107	Gag-pol polyprotein precursor^a^	<0.0001
11	OG0000120	Larval serum protein^b^	<0.0001
12	OG0000134	Glutathione S Transferase^b^	<0.0001
13	OG0000312	Seminal fluid protein^a^	<0.0001

a Gene family expanded in *P. downsi.*

b Gene family contracted in *P. downsi.*

**Figure 4:**
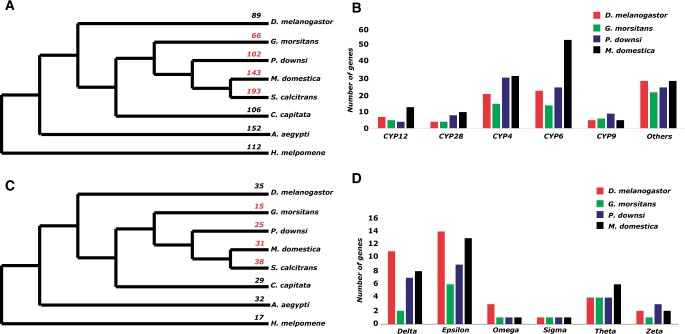
Gene family evolution. (A) Number of genes in Cytochrome P450 gene family across Diptera. (B) Number of genes in various subfamilies of Cytochromes P450 gene family in *G. morsitans*, *P. downsi*, *M. domestica*, and *D. melanogaster*. (C) Number of genes in glutathione *S*-transferase gene family across Diptera. (D) Number of genes in various subfamilies of glutathione *S*-transferase gene family in *G. morsitans*, *P. downsi*, *M. domestica*, and *D. melanogaster.*

One of the gene families identified as significantly expanding or contracting relative to its closest phylogenetic neighbors was cytochrome P450 (CYP450). CYP450 is a diverse superfamily of proteins associated with catabolism and anabolism of xenobiotics and endogenous compounds, that also play a role in insect hormone metabolism (Iga and Kataoka 2012). Overproduction of these detoxification enzymes has possibly allowed numerous insect species to develop insecticide resistance (Scott 1999; Wen *et al.* 2011). *Philornis downsi* and *M. domestica* have an expanded CYP450 gene family in comparison to their most recent common ancestor with *G. morsitans* (family Glossinidae) (Figure  4A). The *P. downsi* CYP450 family is composed of 102 genes in comparison to 66 in *G. morsitans.* An even greater level of expansion was observed in two other members of Muscidae (*M. domestica*: 143 genes and *S. calcitrans*: 193 genes). Compared to *G. morsitans*, the expansion of CYP450 genes is mainly found in CYP4, CYP6, and CYP28 genes (Figure  4B). The CYP6 subfamily in *P. downsi* is composed of 25 genes, almost doubling the number from the 14 genes present in *G. morsitans.* Within *M. domestica* and *S. calcitrans*, some expanded CYP6 and CYP9 genes are clustered along the scaffold (Dermauw *et al.* 2020). We found a similar case in *P. downsi.* For example, there were eight CYP6 genes clustered within the 195-kb region in scaffold 39. We also found an expansion in the CYP4 subfamily from 15 genes in *G. morsitans* to 31 genes in *P. downsi.* A similar expansion was seen in the CYP28 subfamily (four genes in *G. morsitans* to eight genes in *P. downsi*).

Glutathione *S*-transferase (GST) also showed evidence of expansion in *P. downsi*. GSTs are a highly conserved, large family of dimeric enzymes associated with detoxification of endogenous and/or xenobiotic compounds, such as insecticides (Ketterman *et al.* 2011). The *P. downsi* GST family consists of 25 genes and the pattern across the dipterans is similar to that observed within the Cytochrome P450 gene family described above (Figure  4C). For example, in comparison to *G. morsitans* (15 genes), *P. downsi* and *M. domestica* have an expanded number of genes (25 genes in *P. downsi*, 31 genes in *M. domestica*). The GST family is further grouped into six subclasses (Delta, Epsilon, Omega, Sigma, Theta, and Zeta), with Delta and Epsilon being specific subclasses found in the class Insecta. The major expansions of the GST family in *P. downsi* were observed in Delta and Epsilon subclasses (Figure  4D).

We also identified a reduction in size of the Fibrinogen C-terminal Domain-Containing and SCP domain-containing gene families, both known to have immune function properties (Table  1 and Supplementary Table S5). Fibrinogen plays a key role in blood clot formation through the conversion of fibrinogen to insoluble fibrin (Weisel and Litvinov 2017) and the C-terminal domain of fibrinogen is the primary binding site of platelets (Hanington and Zhang 2011). The Sperm-coating glycoprotein (Scp) family contains, among other proteins, antigen 5 (Ag5), which is associated with the venom secretory ducts of stinging insects (Gibbs *et al.* 2008).

The invasion of *P. downsi* has had dramatic negative effects on the endemic avifauna of the Galápagos, including Darwin’s finches. As researchers work to better understand the pathway of invasion and the ecological and evolutionary processes that may have facilitated its invasion to the Galápagos, the need for a high-quality whole genome sequence has grown. The addition of this resource is therefore meant to provide the foundation for further investigations using genomics tools in this system. Gene expression studies could shed light on the observed gene family expansions and contractions noted in our study and present a promising avenue for future research. Further population-scale resequencing of various populations of *P. downsi* will also allow us to explore mechanisms of local adaptation of the parasite to the environment across islands.

## Data availability

The genome assembly has been deposited at DDBJ/ENA/GenBank under the accession JAHXMU000000000. The version described in this paper is version JAHXMU010000000. Short-sequencing reads used for generating this genome have been deposited to NCBI, under bioproject accession number PRJNA747990. Custom scripts and codes used for running all analyses reported in this paper can be found on our lab GitHub page (https://github.com/sangeet2019/Philornis). Supplemental Material available at figshare: https://doi.org/10.25387/g3.14700222.
